# Psychosomatic - psychotherapeutic treatment of stress-related disorders impacts the sphingolipid metabolism towards increased sphingosine and sphingosine-1-phosphate levels

**DOI:** 10.1007/s00406-025-01985-2

**Published:** 2025-03-05

**Authors:** Franziska Werner, Fabian Schumacher, Christiane Mühle, Werner Adler, Caterina Schug, Eva Schäflein, Eva Morawa, Burkhard Kleuser, Johannes Kornhuber, Yesim Erim, Cosima Rhein

**Affiliations:** 1 Department of Psychosomatic Medicine and Psychotherapy, Friedrich-Alexander- Universität Erlangen-Nürnberg (FAU), Erlangen, Germany; 2 Institute of Pharmacy, Department of Pharmacology & Toxicology, Freie Universität Berlin, Berlin, Germany; 3 Department of Psychiatry and Psychotherapy, Friedrich-Alexander-Universität Erlangen-Nürnberg (FAU), Erlangen, Germany; 4 Institute of Medical Informatics, Biometry and Epidemiology, Friedrich-Alexander- Universität Erlangen-Nürnberg (FAU), Erlangen, Germany; 5 Department of Psychotherapy and Psychosomatic Medicine, Faculty of Medicine, Technische Universitat Dresden (TUD), Dresden, Germany

**Keywords:** Psychosomatic medicine, Ceramide, Sphingosine, Sphingosine-1-phosphate, acid Sphingomyelinase, Acid ceramidase, Stress-induced psychiatric disorders, Depression, Post-traumatic stress disorder, Psychotherapy

## Abstract

**Objective:**

Chronic stress is a risk factor for developing stress-induced mental disorders like major depression and post-traumatic stress disorder. Low-grade inflammatory processes seem to mediate this association. The sphingolipid metabolism with its most important lipid messengers ceramide and sphingosine-1-phosphate (S1P) was shown to play an important role in the pathophysiology of affective disorders and inflammation.

**Method:**

We conducted an exploratory trial to investigate the effect of intensive psychosomatic - psychotherapeutic treatment of stress-induced disorders on the biological level. Before and after eight weeks of treatment, blood plasma of 67 patients was analyzed for sphingolipid levels and their metabolizing enzymes. Symptom severity of depression (PHQ-9), anxiety (GAD-7), and somatization (PHQ-15) was assessed in parallel.

**Results:**

During psychosomatic - psychotherapeutic treatment, symptom severity of depression, anxiety, and somatization decreased significantly. Levels of the stress molecule cortisol decreased upon treatment. Enzymatic activities of secreted acid sphingomyelinase (S-ASM) and neutral sphingomyelinase (NSM) increased significantly upon treatment, as well as of neutral ceramidase (NC). Regarding the lipid level, the molar ratio of ceramide species Cer16:0 and Cer18:0 decreased upon treatment, whereas sphingosine and S1P levels increased.

**Conclusions:**

Psychosomatic – psychotherapeutic treatment was associated with a reduction in specific ceramide ratios and an increase in sphingosine and S1P levels potentially resulting from increased activity of sphingolipid metabolizing enzymes. Stress-induced mental disorders might be associated with disturbed sphingolipid levels that seem to be balanced during psychosomatic treatment. This study offers a further piece of evidence that the sphingolipid metabolism could be involved in the pathophysiology of stress-induced disorders, and its analysis could be helpful for treatment monitoring.

## Introduction

Chronic stress is a major burden of modern societies and is associated with several human diseases like cardiovascular disease, autoimmune disorders and psychiatric disorders [1]. Animal studies show that the exposure to chronic stress changes the integrity of the brain with morphologic and functional implications, e.g. regarding higher cognitive functions like learning and memory as well as emotional processing [2]. In a fast reaction to stress, the sympathetic nervous system helps to adapt to the life-threatening situation. However, when stress becomes a chronic situation, the whole organism can be damaged [3]. As a second reaction, the hypothalamic-pituitary-adrenal (HPA) axis with its most important effector cortisol is activated upon stress [4]. Studies revealed a resulting chronic low-grade inflammation that might serve as a mediator between chronic stress and detrimental diseases [5].

Recently, lipid metabolism and, particularly sphingolipids, have been implicated in stress and the pathophysiology of psychiatric disorders. The bioactive sphingolipid central to the rheostat is ceramide [6], which is generated by the hydrolysis of sphingomyelin through the activity of acid sphingomyelinase (ASM), neutral sphingomyelinase (NSM) or alkaline sphingomyelinase depending on the optimum pH of the enzyme [7]. Ceramide can also be generated via *de novo* synthesis [8], by degradation of complex glycosphingolipids [9] or by the salvage pathway that implies the reacylation of the degradation product sphingosine [10]. Ceramide can be degraded to sphingosine and fatty acid by acid and neutral ceramidases (NC). In turn, sphingosine can be phosphorylated by sphingosine kinases 1 and 2 to the other important bioactive sphingolipid sphingosine-1-phosphate (S1P), balancing the tightly regulated rheostat. Increases in ceramide levels were found to be associated with cell death, cell differentiation, senescence and autophagy, whereas increases in S1P were shown to relate to cell survival, inflammation, and cell migration [6].

The impact of stress on ceramide levels has been identified in rodent models. Recent work in rats demonstrate that the application of exogenous corticosterone (which resembles cortisol in humans) resulted in an increase in ceramide levels in the brain. The same effect was observed when rats were treated with chronic unpredictable stress [11]. Similarly, a paradigm inducing psychosocial stress in mice led to an increase in ceramide levels and tumor necrosis factor alpha [12]. Moreover, it was shown in mice that ceramide-enriched exosomes were released by NSM-2 into the blood plasma upon stress and mediated stress-induced MDD [13].

Several human studies found increased ceramide levels in patients suffering from stress-related psychiatric disorders like major depressive disorder (MDD), post-traumatic stress disorder (PTSD) and anxiety disorders. In patients experiencing a major depressive episode, ASM showed increased enzymatic activity in peripheral blood cells compared with healthy controls [14]. Blood plasma levels of several ceramide species were increased in patients suffering from MDD compared with healthy controls [15]. This was confirmed in a large study on patients with MDD and bipolar disorder that identified increased plasma ceramide Cer16:0, Cer18:0, Cer20:0, Cer22:0, Cer24:0 and Cer24:1 levels compared with controls [16], and in a small study with MDD patients [17]. The severity of depression symptoms was associated with higher plasma levels of ceramide Cer16:0 and Cer18:0 in patients with coronary artery disease [18].

Accordingly, sphingomyelin (SM) levels in plasma were found to be decreased in MDD patients [19, 20], and the SM23:1/SM16:0 ratio was negatively correlated with the severity of depressive symptoms [21]. In veterans suffering from PTSD, the secreted form of ASM exhibited increased activity levels, and Cer18:0 and S1P levels in blood plasma were increased compared with controls [22]. A recent study on 235 PTSD patients and 241 healthy controls found significant differences in ceramide and sphingomyelin levels between both groups [23]. In patients suffering from anxiety disorders like fear, avoidance of perceived threats or panic attacks, an inverse correlation was found between anxiety symptoms and the plasma ratios SM23:1/SM16:0 and phosphatidylcholine (PC) *O*-36:4/ Cer20:0 [21]. A specific sphingolipid, *N*-(hexadecanoyl)-deoxysphing-4-enine-1-sulfonate, was also negatively correlated with anxiety symptoms in patient plasma of a recent study [24]. Several animal studies were conducted on anxiety-like behavior and sphingolipids. It was shown that infusion of C16:0 ceramide into the basolateral amygdala resulted in anxiety-like behavior in mice [25]. In a mouse model for social fear with comorbid depression, the activity of sphingomyelinases and ceramidases as well as the sphingolipid levels and sphingolipid ratios were altered in several brain regions [26]. In the prefrontal cortex of mice characterized by anxiety-like behavior a decrease in the expression of alkaline ceramidase 2 was observed [27]. The decrease of ceramide synthase 1 and subsequently C18:0 gangliosides decreased the risk of developing anxiety-like behavior [28]. Stress-induced anxiety was reported to increase TNF-a levels and to reduce the expression of the S1P receptor 2 in hippocampal tissue of rats which affects immune cell trafficking, among others [29].

Treatment of stress-induced disorders includes psychotherapeutic therapy and pharmacologic medication. How state-of-the-art treatment changes the sphingolipid profile is hardly investigated. In our study we found a decrease in the mRNA expression of *SMPD1*, coding for ASM, in untreated MDD patients upon treatment with antidepressant medication [30]. Cell culture studies show that some antidepressant drugs, named FIASMAs, inhibit the enzymatic activity of lysosomal ASM [31]. However, the decrease in ASM activity could not be found in plasma after the treatment of MDD patients with antidepressant drugs [32]. Further, a clinical study investigating the treatment effects on the sphingolipid level observed that patients receiving antidepressants had higher levels of Cer18:0, Cer22:0 and Cer24:0 than patients not receiving those drugs [16], maybe due to an inhibitory effect of antidepressants on acid ceramidase [33].

However, no study exists how psychotherapeutic treatment in contrast to pharmacological treatment affects the sphingolipid metabolism. In this study, we aimed at investigating the impact of psychosomatic- psychotherapeutic treatment of patients suffering from stress-related disorders on sphingolipid metabolism. This work will help to better understand the role of sphingolipids in stress-related mental disorders.

## Materials and methods

### Study sample

We conducted an exploratory study and invited all patients who were enrolled at the inpatient and daycare unit of the Department of Psychosomatic Medicine and Psychotherapy at the University Hospital Erlangen, Germany, between September 2019 and March 2020 for participation. Inclusion criteria were age ≥ 18 years, sufficient German language proficiency, and regular admission for an inpatient or daycare unit treatment. Exclusion criteria were acute psychotic disorder, degenerative brain disorders (e.g. Alzheimer’s disease), acute suicidality, and current substance dependency. This strategy resulted in the inclusion of 67 individuals (54 females, 13 males, average age 39.6 years ± 14.5, range from 21 to 67 years). During the first two days of enrollment, a structured clinical diagnostic interview was conducted by trained psychologists. Before starting treatment and at the last day of enrollment, patients completed the psychometric assessments using an electronic system, and blood was drawn for biological analyses. Patients received intensive psychosomatic-psychotherapy treatment for eight weeks including an adjustment of psychotropic drug administration if appropriate. About half of patients received antidepressant medication (53.6%). Most patients received a monotherapy of selective serotonine reuptake inhibitors (SSRI, *N* = 8), a monotherapy of selective serotonine and norepinephrine reuptake inhibitors (SSNRI, *N* = 5), or a monotherapy of noradrenergic and specific serotonergic antidepressants (NaSSA, *N* = 2). Five patients took a combination of a SSRI/SSNRI or NaSSA and a tricyclic antidepressant agent, and 2 patients took a combination of a norepinephrine-dopamine reuptake inhibitor and a SSNRI or NaSSA. Anti-inflammatory medication was applied to 21.4% of patients. 12 patients received anti-inflammatory drugs (NSAIDs, corticosteroids, monoclonal antibodies, or mesalazine) during the observation period (Table 1). 11 patients did not indicate their medication. This inpatient and daycare psychotherapeutic treatment is special for Germany, and health insurances reimburse expenses. Only those individuals who participatedat both time points were included in the analysis in order to provide longitudinal data. The study was approved by the Ethics Committee of the Friedrich-Alexander-University Erlangen-Nürnberg (FAU, ID 200_19 Bc) and conducted in accordance with the Declaration of Helsinki. Written informed consent was obtained from all participants.

**Table 1:** **Socio-demographic and clinical characteristics of included patients.** NSAIDs, nonsteroidal anti-inflammatory drugs, SSRI, selective serotonine reuptake inhibitors, SSNRI, selective serotonine and norepinephrine reuptake inhibitors, NaSSA, noradrenergic and specific serotonergic antidepressants

Demography	
Number of patients	*N* = 67
Age (Mean, SD, range)	39.6 ± 14.5, 21–67
Sex (% female)	80.6%
Patients with antidepressant medication	53.6%
SSRI antidepressant monotherapy	*N* = 8
SSNI antidpressant monotherapy	*N* = 5
NaSSA antidepressant monotherapy	*N* = 2
SSRI/SSNRI/NaSSA plus tricyclic antidepressant	*N* = 5
NDRI plus SSNRI or NaSSA	*N* = 2
Patients with anti-inflammatory medication	21.4%
NSAIDs	*N* = 8
Corticosteroids	*N* = 2
Monoclonal antibodies + corticosteroids	*N* = 1
Monoclonal antibodies + mesalazine	*N* = 1
**Diagnosis**	
Depression (F31, F32, F33, F34)	*N* = 54 (80.6%)
Anxiety, Phobia, Compulsive disorder (F40, F41, F42)	*N* = 51 (76.1%)
Posttraumatic stress disorder (F43.1)	*N* = 32 (47.8%)
Somatization (F45)	*N* = 27 (40.3%)
Eating disorder (F50)	*N* = 19 (28.4%)
One mental disorder diagnosis	*N* = 8 (11.9%)
Two mental disorder diagnoses	*N* = 23 (34.3%)
Three mental disorder diagnoses	*N* = 20 (29.9%)
Four or more mental disorder diagnoses	*N* = 5 (7.5%)

Diagnoses were assessed using MINI-DIPS according to ICD-10

### Blood collection and cortisol measurement

Blood was collected at 8 a.m. in the morning after overnight fasting to minimize circadian and nutritional effects. To obtain blood plasma, whole blood was collected into EDTA-containing vials (Sarstedt, Germany), centrifuged for 10 min at 2000× *g* at room temperature, aliquoted, and stored at − 80 °C for later assays. Samples were processed within two hours. Cortisol was quantified at the Central Laboratory of the Universitätsklinikum Erlangen, Germany (DIN EN ISO 15189 accredited) from separately collected vials using an electro-chemiluminescence immune assay (Elecsys Cortisol II Kit) on a cobas e801 device (Roche, Switzerland).

### Psychometric assessment

For obtaining a standardized diagnosis, trained psychologists conducted the structured clinical interview MINI-DIPS (*Diagnostisches Interview bei psychischen Störungen*, in German only). To assess the symptom severity of mental disorders before and after treatment, we conducted the German version of the Patient Health Questionnaire (PHQ-D, *Gesundheitsfragebogen für Patienten*; [34]) that includes subscales to assess symptom severity of depression (PHQ-9), somatization (PHQ-15) and generalized anxiety (GAD-7) [35] using an electronic system.

### Psychosomatic treatment

Patients received a multimodal group-based therapy [35, 36]. Techniques of both cognitive behavioral and psychodynamic psychotherapy were applied. Weekly psychotherapeutic elements were psychotherapy in individual (1 × 50 min) and group (2 × 100 min) format, skills training (2 × 60 min), mindfulness and relaxation methods (2 × 50 min), art therapy (1 × 120 min), Concentrative Movement Therapy (1 × 120 min), and psychopharmacological therapy, if appropriate. Standardization and quality of treatment was controlled by weekly team meetings and internal and external supervision of the whole therapeutic team.

### Enzyme activity assays for sphingomyelinases and ceramidase

The activity of sphingolipid enzymes was quantified in blood plasma using fluorescent substrates, BODIPY-FL-C12-Sphingomyelin (D-7711, Invitrogen, Carlsbad, CA, USA/Life Technologies, Grand 15 Island, NY, USA) for secreted ASM (S-ASM) [37] and NSM [38] and NBD-C12-ceramide (Cay10007958-1, Cayman Chemical, via Biomol GmbH, Hamburg, Germany) for NC as described previously [37]. Briefly, the reaction was performed in 96 well polystyrene plates with 58 or 50 pmol fluorescently labelled SM or Cer, respectively, in a buffer mix totaling 50 µl in volume. The reaction was initiated by the addition of 6 µl of a 1:10 dilution of plasma in physiological 154 mM NaCl solution. After incubation at 37 °C for 6–48 h depending on the enzyme, reactions were stopped by freezing at − 20 °C and stored until further processing. For direct chromatography, 1.5 µl of the reaction was spotted directly without further purification on silica gel 60 thin layer chromatography plates (ALUGRAM SIL G, 818232, Macherey-Nagel, Düren, Germany). Product and uncleaved substrate were separated using ethyl acetate with 1% (v/v) acetic acid as a solvent for all enzymes. Spot intensities were detected on a Typhoon Trio scanner and quantified using the ImageQuant software (GE Healthcare Life Sciences, Buckinghamshire, UK). All enzyme activity assays were carried out with four replicate dilutions of each sample and using the same lot of reagents and consumables and performed by a single operator.

### Sphingolipid quantification by liquid chromatography tandem-mass spectrometry (LC-MS/MS)

Blood plasma (20 µl) was subjected to lipid extraction using 1.5 ml methanol/chloroform (2:1, v/v) [39]. The extraction solvent contained d_7_-sphingosine (d_7_-Sph), d_7_-sphingosine-1-phosphate (d_7_-S1P), ceramide C17:0 (Cer17:0) and sphingomyelin C16:0-d_31_ (SM16:0-d_31_) (all Avanti Polar Lipids, Alabaster, USA) as internal standards. Sample analysis was carried out by liquid chromatography tandem-mass spectrometry (LC-MS/MS) using a 1260 Infinity HPLC coupled to a 6490 triple-quadrupole mass spectrometer (both Agilent Technologies, Waldbronn, Germany) operating in the positive electrospray ionization mode (ESI+). Analytes were measured by multiple reaction monitoring (MRM) as described [40]. Quantification of Cer and SM sub-species (fatty acyl chain lengths: 16:0, 18:0, 20:0, 22:0, 24:0 and 24:1), as well as Sph and S1P was performed with MassHunter Software (version 10.1, Agilent Technologies). Sphingolipid plasma levels are expressed as “pmol/20 µl” or molar ratios.

### Statistical analysis

We examined changes between both time points using paired t tests or Wilcoxon matched-pairs signed rank test after testing for normality of differences. ANOVA with repeated measures was applied when co-factors were included. We corrected for multiple testing using the Benjamini-Hochberg method [41], corrected for 15 tests and report corrected p values in the results section. The significance level was set to 0.05. A statistical trend was defined for significance levels up to *p* = 0.1. Figures show mean values and standard deviation (SD). Statistical analyses were performed using R V 4.2.0 [42], GraphPad Prism, and IBM SPSS Statistics version 21.

## Results

### Symptom severity decreases upon psychosomatic treatment

**Fig. 1:**
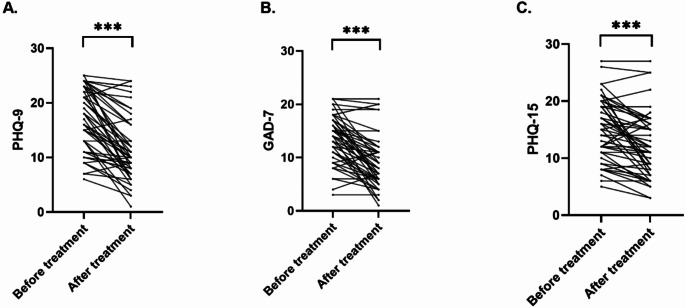
Symptom severity decreases upon psychosomatic treatment. Paired t tests revealed a significantly reduced symptom severity regarding depression indicated by PHQ-9, anxiety indicated by GAD-7, and somatization indicated by PHQ-15 when comparing psychometric values before and after treatment. Bars indicate mean values with SD, *** *p* < 0.001

The analysis regarding the effect of psychosomatic treatment on symptom severity revealed a significant amelioration of main symptoms. Paired t tests showed significantly reduced symptom severity regarding depression (from 17.02 ± 5.70 to 11.26 ± 5.90) indicated by PHQ-9 (*p* < 0.001, Paired t test; Fig. 1A), anxiety (from 13.84 ± 4.60 to 9.44 ± 5.40) indicated by GAD-7 (*p* < 0.001, Paired t test; Fig. 1B), and somatization (14.96 ± 5.40 to 12.06 ± 5.70) indicated by PHQ-15 (*p* < 0.001, Paired t test; Fig. 1C) when comparing psychometric values before and after treatment (*n* = 67). Antidepressant or anti-inflammatory medication did not impact psychometric changes.

### Activities of sphingolipid metabolizing enzymes increase upon psychosomatic treatment

**Fig. 2:**
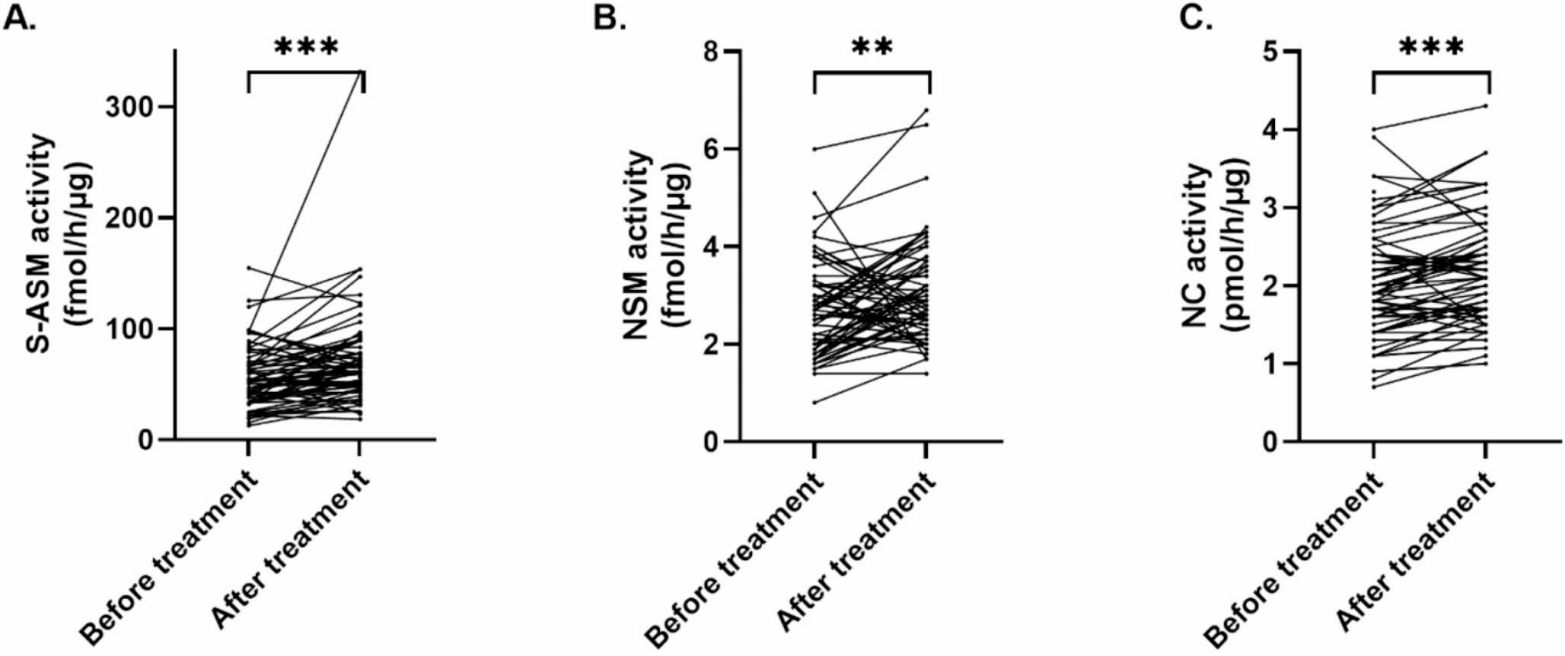
Activities of sphingolipid metabolizing enzymes increase upon psychosomatic treatment. Significantly increased enzymatic activities of S-ASM, NSM, and NC were observed when comparing values before and after treatment. Enzymatic activities were measured using fluorescently labelled substrate and thin-layer chromatography. Bars indicate mean values with SD, ** *p* < 0.01, *** *p* < 0.001. ASM, NSM: Acid and neutral sphingomyelinases; NC: Neutral ceramidase

Three enzymes regulating ceramide levels were analyzed in plasma samples before and after psychosomatic therapy. Respective paired tests after testing for normality of differences revealed an increase in enzymatic activities of S-ASM, NSM and NC in blood plasma of patients (*n* = 63, Fig. 2). S-ASM mean values before treatment (56.0 fmol/h/µg ± 28.10) increased to 71.70 fmol/h/µg ± 45.70 (*p* < 0.001, Wilcoxon matched-pairs signed rank test; Fig. 2A). Regarding NSM, levels increased from 2.68 fmol/h/µg ± 0.10 to 3.08 fmol/h/µg ± 1.03 (t = 2.969, *p* = 0.003, Paired t test; Fig. 2B). Mean values of NC increased from 2.01 pmol/h/µg ± 0.73 to 2.17 pmol/h/µg ± 0.70 (*p* < 0.001, Wilcoxon matched-pairs signed rank test; Fig. 2C). Antidepressant or anti-inflammatory medication did not impact changes in enzymatic activities.

### The molar ratio of ceramide species C16:0 and C18:0 decreases upon psychosomatic treatment

**Fig. 3:**
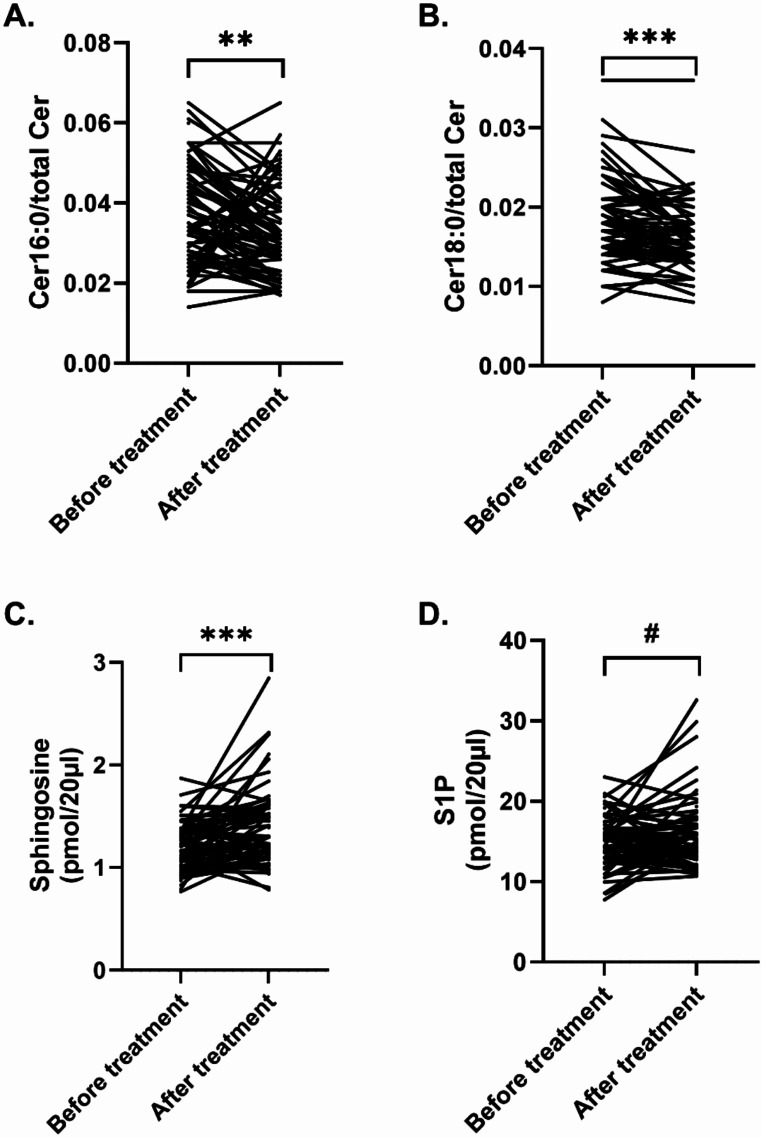
Relative levels of ceramide species decrease upon psychosomatic treatment, whereas sphingosine and S1P levels increase. Significantly decreased molar ratios of ceramide C16:0 (Cer C16:0/total ceramides) and ceramide C18:0 (Cer C18:0/total ceramides) upon treatment was observed. Sphingosine levels increased significantly. S1P levels also increased upon treatment (*p* = 0.1). Sphingolipid levels were measured using LC-MS/MS. Bars indicate mean values with SD, ** *p* < 0.01, *** *p* < 0.001, ^#^*p* = 0.1

Sphingomyelinases hydrolyze sphingomyelin to ceramide. The molar ratio of ceramide C16:0 (Cer16:0/total ceramides) decreased in patients (*n* = 62) upon treatment from 0.037 ± 0.013 to 0.033 ± 0.012 (*p* = 0.003, Wilcoxon matched-pairs signed rank test; Fig. 3A). The molar ratio of ceramide C18:0 (Cer18:0/total ceramides) decreased from 0.018 ± 0.005 to 0.016 ± 0.005 (*p* < 0.001, Wilcoxon matched-pairs signed rank test; Fig. 3B). Molar ratios of ceramide species Cer20:0, Cer22:0, Cer24:0 and Cer24:1 did not show significant changes upon treatment. Antidepressant or anti-inflammatory medication did not impact changes in ceramide ratios.

### Sphingosine levels increase upon psychosomatic treatment

Sphingosine levels, the metabolite emerging from ceramide hydrolysis by ceramidases, increased in patients after treatment (*n* = 62) from 1.19 pmol/20 µl ± 0.24 to 1.39 pmol/20 µl ± 0.38 (*p* < 0.001, Wilcoxon matched-pairs signed rank test; Fig. 3C). Antidepressant or anti-inflammatory medication did not impact sphingosine changes.

### S1P levels increase upon psychosomatic treatment

**Fig. 4:**
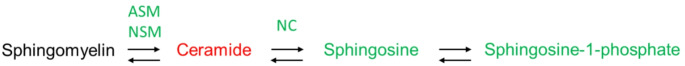
Schematic of changes in the ceramide/S1P rheostat upon psychosomatic treatment. Indicated are only those enzymes and sphingolipids that were analyzed in this study. Increased values after treatment are indicated in green, decreased ratio values in red. ASM, NSM: Acid and neutral sphingomyelinases; NC: Neutral ceramidase

S1P is the product of the phosphorylation of sphingosine. A statistical trend indicating an increase in S1P levels in patients emerged when comparing pre and post treatment values (*n* = 62; 14.80 pmol/20 µl ± 3.21 vs. 15.80 pmol/20 µl ± 4.49, t = 1.691, *p* = 0.1, Paired t test; Fig. 3D). A stratified analysis showed that medication did not impact S1P changes. Figure 4 gives a schematic of changes in the ceramide/S1P rheostat upon psychosomatic therapy.

### Cortisol levels decrease upon psychosomatic treatment

**Fig. 5:**
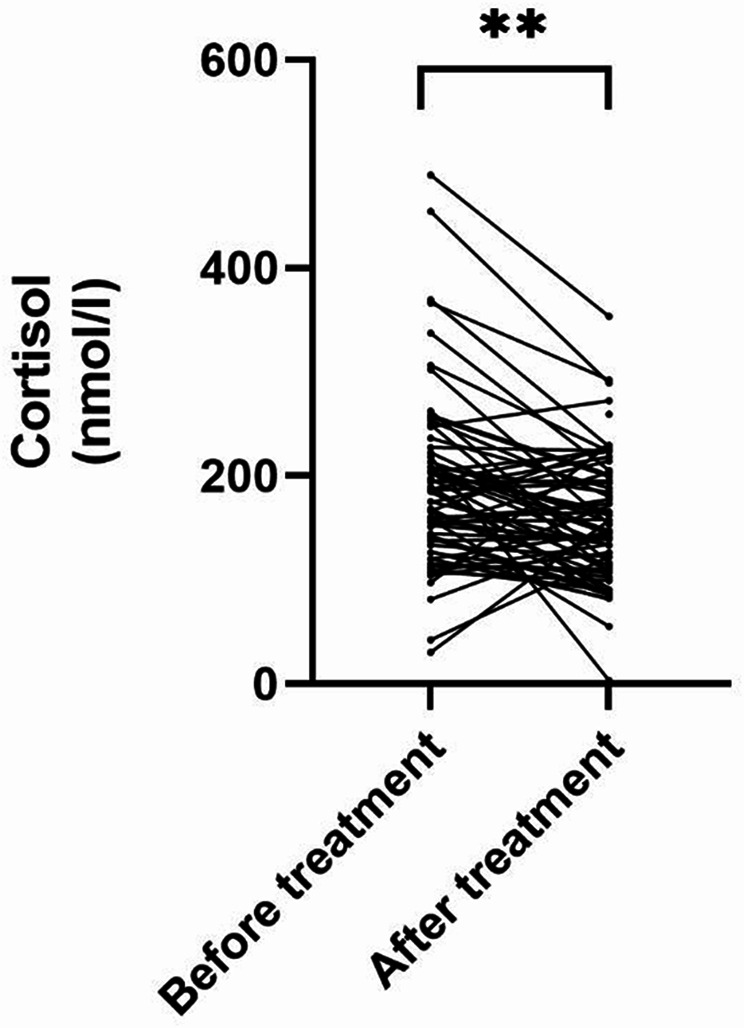
Cortisol levels decrease upon psychosomatic treatment. Paired t test showed a significant decrease of cortisol levels when comparing values before and after treatment. Bars indicate mean values with SD, ** *p* < 0.01

To identify effects of changes in sphingolipid metabolism on immune function, we analyzed cortisol levels as an indicator of the HPA axis. Cortisol levels decreased significantly when comparing values before (189.40 nmol/l ± 84.02) and after (157.10 nmol/l ± 61.47) treatment (t = 3.84, *p* = 0.002, Paired t test; Fig. 5).

## Discussion

Our study showed for the first time that intensive psychosomatic – psychotherapeutic treatment of stress-related disorders is associated with changes in the sphingolipid rheostat, namely a decrease in the ratio of specific ceramide subspecies, and an increase in sphingosine and S1P levels. Ceramides are associated with cellular stress, whereas S1P is the counterpart of ceramide in the rheostat and associated with cellular growth and survival. In the current study, treatment resulted in an increase in the two sphingomyelinase activities that are supposed to increase ceramide levels, but, interestingly, a decrease in the ratio of two ceramide species was detected. This seems contradictory if only a small aspect of the sphingolipid rheostat is investigated. As also an increase in ceramidase activity was observed, ceramide generated by sphingomyelinases seems to be metabolized to sphingosine, with a subsequent phosphorylation to S1P. Thus, the rheostat might be disturbed in stress-associated diseases and will be rebalanced by treatment into the direction of sphingosine and S1P.

MDD was the most prevalent diagnosis in our study. Our earlier studies investigated treatment effects of antidepressant medication on ASM activity. In cell culture studies, a significant effect of FIASMA treatment on lysosomal ASM activity was observed [31]. After pharmacological treatment of naïve MDD patients, ASM was found to be decreased, but at the mRNA level [30]. No effects of antidepressant treatment on S-ASM activity and sphingolipid levels could be detected in a clinical study [32]. However, one clinical study investigating the treatment effects on the sphingolipid level observed that MDD patients receiving antidepressants had higher ceramide levels than patients not receiving those drugs [16]. Our results are in line with this and add a further piece of evidence that ceramide levels are impacted by MDD treatment. However, diverging study results might relate to different kinds of therapies. To date it is not clear if psychopharmacological medication and psychotherapy affect the same molecular targets. Also the sub-classification of MDD types might be necessary to address distinct molecular pathways. Suffering from multiple comorbidities could also be relevant for treatment outcomes. Thus, the direction of sphingolipid change by MDD treatment seems to be highly affected by type of treatment, diagnosis, comorbidity, and possibly further variables. More studies are needed to pinpoint the treatment effect of stress-induced mental disorders on ceramide and its metabolizing enzymes.

Chronic low-grade inflammation has been discussed as a mediator between chronic stress and detrimental diseases [5]. The underlying hypothesis describes that psychosocial stress induces the generation of inflammasomes, which affect the immune-to-brain-communication via the humoral, neural, or cellular route, and in turn the neurotransmitter release and reuptake [43]. Only few studies investigated the abnormalities in peripheral immune cells in stress-induced disorders. PTSD [44] and MDD [45] have been found to be associated with a decrease in regulatory T cells, which might result in an overshooting of the inflammatory response. MDD was shown to be associated with premature T cell ageing [46]. Regarding the effects of treatment, the pharmacological inhibition of ASM activity by FIASMAs in patients suffering from MDD has been found to increase regulatory T cells both in vivo and in vitro [47]. A further study confirmed the increase in regulatory T cells upon treatment of MDD patients [48]. However, a different study found a decrease in regulatory T cells after treatment of MDD for 8 weeks, but it has to be taken into account that patients were not untreated when entering the study, which might have affected results [49]. S1P has been shown to be an important regulator of immune function. Several studies demonstrate the essential role of S1P and its receptors in immune cell development, differentiation, and trafficking, and in signaling actions during inflammatory processes [50]. One of the enzymes generating S1P, SK1, has been proven to play an important role in the inflammatory response in cellular models, especially via the TNFα signaling pathway [51]. The S1P receptor 3 (S1P3) was suggested to promote stress resilience, as its overexpression in the murine prefrontal cortex reduced TNFα-mediated inflammatory processes and resulted in a resilient phenotype, while its knock-down increased anxiety- and depression-like behaviors. In addition, the mRNA expression of S1P receptor 3 was decreased in PTSD patients and increased in resilient individuals [52]. Accordingly, the increase in S1P levels after treatment could rebalance the dysregulated immune status in stress-related disorders. However, S1P is also known for its pro-inflammatory effect. This could relate to a negative feedback mechanism of S1P circulating in the blood and the expression of S1P3 receptors in brain tissue: higher receptor density in the prefrontal cortex could result in a lower pro-inflammatory S1P load in the blood. As the present study shows a significant increase in sphingosine and S1P after psychosomatic therapy, the receptor density in the prefrontal cortex might initially decrease as an acute reaction within the first eight weeks and rebalance as a long term effect. Thus, S1P and its receptors could play a regulatory role regarding immune activation and inflammation in stress-related psychiatric diseases. Regarding immune parameters, our results show a decrease of cortisol during treatment. This could resemble the decrease in stress experience after treatment and changed behavior regarding stress management. In parallel, ceramide C16 ratio decreased during treatment, which is known to be activated during cellular stress. However, the interplay between sphingolipids and the HPA axis is very complex, and results are partly conflicting. Mutations in the gene coding for sphingosine-1-phosphate lyase (*SGPL1*), the enzyme that hydrolyzes S1P irreversibly, results in the SGPL1 insufficiency syndrome (SPLIS), a multi-systemic disorder with incorporation of steroid-resistant nephrotic syndrome and primary adrenal insufficiency. Knockout of *SGPL1* in human adrenocortical (H295R) cells, resulting in increased S1P levels, abrogated cortisol production. Wild-type SGPL1 was able to rescue cortisol production in this in vitro model of adrenal disease [53]. However, this was shown only for a short time course. Long term effects of increased S1P levels on cortisol secretion are still under debate. Further, S1P was found to stimulate cortisol secretion, as cortisol can activate the expression of the acid ceramidase gene (*ASAH1*) [54] with subsequent ceramide degradation and sphingosine increase [55]. This points to an intra-adrenal feedback mechanism that ensures optimal steroid hormone output. Also in this case, only short term effects were measured [56]. A further study showed that sphingosine and S1P were able to stimulate cortisol biosynthesis in H295R cells, with also only short term effects, as cortisol levels reached again controls after 12 h [57]. Together, these studies on sphingolipids and cortisol identified a novel mechanism by which cortisol secretion is regulated via S1P in the human adrenal cortex. To pinpoint the interplay in the long run and during treatment needs the conduction of further studies.

Limitations are that not all metabolites and enzymes of the rheostat could be analyzed due to the lack of methodology. Assays for lacking enzyme activities should be developed to assess the complete rheostat. Diagnosis groups were too small to detect diagnosis-specific treatment effects. Further studies should stratify for different diagnoses. As 80% of patients were female, the results might not be generalizable for men.

Taken together, this is the first study that analyzes the most important metabolites in the sphingolipid rheostat during psychosomatic - psychotherapeutic treatment of stress-related mental disorders. The association of treatment with decreased ceramide and increased sphingosine/S1P levels offers a novel piece of evidence that sphingolipids might be involved in the pathophysiology of stress-related disorders.
